# Medicine

**Published:** 1899-03

**Authors:** Theodore Fisher


### Progress of the Medical Sciences.

---

### MEDICINE.

At the present time the **treatment and prevention of tuberculosis** occupies considerable attention. To pass under notice in this short review the various points bearing upon the subject brought into prominence during the past few months would possibly not be very profitable. It is well, however, sometimes to use our judgment concerning apparently well-established facts, and to consider whether there may not be sources of error causing what appears to be fact to be mixed with fallacy. The members of the medical profession are the leaders of public thought in these matters, and if we lay down the law dogmatically at one time and a few years later formulate rules of different character, we give good reason for those who look to us for guidance to treat our opinions with scant courtesy.

Much blame has been thrown upon **milk**, by laboratory workers and other observers, as a source of infection of tuberculosis in children. Dr. Delépine[1] prefaces his valuable lecture upon tuberculosis and the milk-supply with some rather strong remarks. He says: "The difficulty of dealing with tuberculosis is further increased by the general ignorance of the public. The danger which they incur from a contaminated milk-supply is appalling in its magnitude, yet because this danger is of an insidious nature and its effects are often so gradual that it is difficult to trace their onset they prefer to have their quietude not disturbed. They are also afraid that their purses might have to be opened. Rather than face such possibilities they prefer to believe the few experts who still hold views which numerous most searching investigations have proved to be wrong."

Dr. Delépine's investigations certainly appear to give cause for alarm. He found that of 143 samples of milk 25 gave rise to tuberculosis in guinea pigs. The experiments of Drs. Kanthack and Sladen[2] are perhaps still more striking. Of 90 guinea pigs, inoculated with milk from the dairies in Cambridge, 23, that is 25.55 per cent., died. Dr. Delépine strengthens his attack upon milk by quoting the Registrar's returns of the causes of death at different ages in London, which assign forms of tuberculosis, other than phthisis, to 1,232 cases under the age of one year; whereas later in life, even for so long a period as that between the ages of five and twenty years, there are only 474 in which death is certified as due to these forms of tuberculosis.

Sir R. Thorne Thorne[3] also lays great stress upon the

[1] *Lancet*, 1898, ii. 733. [2] *Ibid.*, 1899, i. 75. [3] *Ibid.*, 1898, ii. 1289.

Registrar's statistics. He mentions that of recent years the death-rates from most varieties of tuberculosis have greatly diminished, but that "when the death-rates from tabes mesenterica, a form of tuberculosis in which the infection is received into the alimentary canal instead of into the lungs were examined, it was found not only that all the gain attained at other ages had been lost in the case of children and infants, but that in addition to this there had been a very heavy increase in deaths from this cause amongst infants under one year of age." Sir R. Thorne Thorne further states that "this increase had gone hand in hand with a steady increase in the consumption of cow's milk as a food in this country," and that "the danger to man, and especially to the infant population, was one of real gravity, and the loss of child-life due to this disease in milch-cows was appalling."

This is a heavy indictment of milk, and it will be valuable to see what proofs of the truth of the accusation are furnished by the *post-mortem* room. It may be well, however, to pause for a moment to consider peculiarities in the **modes of infection** of children by the tubercle bacillus.

In the adult, as is well known, the apex of the lung is the spot in the body most likely to be first attacked. In the child that is not so. Ordinary phthisical disease of the lung spreading from above downwards is decidedly rare. Infection of the lung is common enough, but the disease enters the lung by a somewhat circuitous course. Tubercle bacilli finding entrance to the lungs are taken up by the lymphatics, and are carried to the bronchial and mediastinal lymph glands. In these glands the disease advances; they enlarge and become caseous, and sooner or later either local invasion of the lung, or general tuberculous infection of the body, usually takes place. Tubercle bacilli finding entrance to the intestines also generally fail to give rise to local lesions. They are first stopped by the mesenteric glands, and finding a suitable soil there may give rise to enlargement and caseation, as in the case of the bronchial glands. Ulcers in the intestines may occur, but when present they are usually of much more recent date than the associated disease of the mesenteric glands.

If the indictment against milk be correct, the *post-mortem* room will furnish us with numerous examples of disease of the mesenteric glands, at the time of life in which children are drinking large quantities of cow's milk. Dr. Sims Woodhead[1] was the first to write upon this subject, and from examination of 127 cases of tuberculosis in children, he found the mesenteric glands caseous in no less than 100 cases. The mediastinal glands were also, however, caseous in 95 cases; but in most of those Dr. Woodhead considered the disease more recent in the thorax than in the abdomen. These statistics taken alone would appear to put the subject beyond the region of dispute.

[1] *Rep. Lab. Roy. Coll. Phys., Edinb.*, 1889, i. 179.

Other observers, however, have not met with the same localisation of tuberculosis of the lymph glands as Dr. Woodhead. Dr. Carr[1] found 79 cases in which tuberculosis in children appeared to start in the thoracic glands, against 20 in the abdominal; and Dr. Colman,[2] after examination of *post-mortem* records at the Children's Hospital, Great Ormond Street, expressed the opinion that, while he did not doubt the possibility of infection of milk in some cases, he was led to attach much more importance to the condition of the thoracic lymphatic glands, as the process was more advanced in them, as a rule, than in the mesenteric glands, and in several they were extensively caseous, when the mesenteric glands showed little or no change.

Dr. Carr and Dr. Colman are not alone in their opinion. Kempner[3] expresses the opinion that tuberculosis, in childhood especially, originates from disease of the bronchial glands. Comby[4] considers that the respiratory tract is always the starting point. In 28 necropsies on children dying of tuberculosis, under the age of two years, the mediastinal glands were affected in every instance. Kossel[5] in 14 necropsies found caseous bronchial glands in 10 cases, and the mesenteric caseous in one; and Weiderhofer[6] found intestinal tuberculosis in only 8 per cent. of cases of tuberculosis in children under the age of two years. But the most striking experience is that of Dr. Emmett Holt.[7] In 119 necropsies upon children dying of tuberculosis, he found caseous bronchial glands in no less than 108, and caseous mesenteric glands in only 38; and in nearly every case of the latter, he considered the abdominal disease had followed the thoracic. He believes that the importance of infection by the intestine has been "greatly exaggerated," and in his opinion does not occur in more than 1 or 2 per cent. of the cases. Dr. Holt quotes Northrup as having had a similar experience to his own. In necropsies on 125 cases of tuberculosis in children, only one case was found in which the disease appeared to have started in the alimentary tract, while in 88 it was clearly through the bronchial lymph glands.

At the Bristol Royal Infirmary the *post-mortem* examinations upon children are not numerous, but those seen during the time I have had charge of the pathological department afford little evidence of primary infection by the alimentary tract. Of ten children of the age of two years and under dying of various forms of tuberculosis, in nine the bronchial and mediastinal glands were caseous. In four of these the mesenteric glands showed a few caseous points, but the disease was much less advanced than in the bronchial glands. In one case, however,

[1] *Lancet*, 1894, i. 1177. [2] *Brit. M. J.*, 1893, ii. 740.
[3] *Münch. med. Abh.*, *j. Reihe*, Heft 17; abstract in *Brit. M. J.*, 1896, ii. Epitome, p. 52.
[4] *Klin. Therap. Woch.*, 1898, v. 604; abstract in *Pediatrics*, 1899, vii. 44.
[5] *Kinder-Arzt.*, 1896, vii. 19; abstract in *Pediatrics*, 1896, ii. 356.
[6] Quoted in *Med. News*, 1896, lxix. 609.
[7] *Med. News*, 1896, lxix. 656.

the mesenteric glands were alone affected; but this was the only case, not only under the age of two years, but at any age, seen in the *post-mortem* room during the past four and a half years, in which infection appeared to have taken place through the alimentary tract.

Another mode of entrance is by the **tonsils**. I have had one well-marked instance of that mode of infection, and some of the statistics quoted above give others; but since the tonsils are open to infection by either the air or milk, examination of such cases has no bearing upon the subject before us.

To return to the question of **infection by the alimentary canal**, the above *post-mortem* statistics must have made it clear that the Registrar's statistics, upon which Dr. Delépine and Sir R. Thorne Thorne laid so much stress, must be, in very great degree, fallacious. The *post-mortem* room shows disease of the mesenteric glands unassociated with tuberculous disease elsewhere to be distinctly rare, consequently tabes mesenterica cannot be of the "appalling" frequency those statistics make it appear to be.

Dr. Carr[1] refers to this point. He says that he has notes of nearly 100 *post-mortem* examinations of cases that clinically might have been called tabes mesenterica, yet in only one of the 100 was any abdominal tuberculosis present. Tuberculosis of the mesenteric glands, however, does exist; but when we find it present, it is not necessary to conclude that the infection was conveyed by milk. In the majority of cases it is secondary to infection of the respiratory tract.

In those cases in which the disease in the mesenteric glands is much less advanced than in the bronchial glands, infection obviously takes place from tubercle bacilli conveyed to the intestines by swallowed infected bronchial secretion. Our experience of early phthisis in adults shows us how the bronchial secretion of bronchitis that precedes local disease may swarm with tubercle bacilli. When tubercle bacilli find their way into the bronchial tubes of children, one can easily understand how, multiplying there and causing increased secretion, the number of bacilli that are absorbed by the lymphatics and enter the bronchial lymph glands must be small, compared with those that, coughed into the mouth in bronchial mucus, are swallowed and finally enter the intestines. There are, however, not only a few cases in which the disease is more advanced in the mesenteric glands than in the bronchial, but occasionally a case occurs in which the mesenteric glands are alone affected. I am not prepared to admit, however, that even these cases are the result of infection derived from cows.

The milk from which babies are fed no doubt frequently stands for the greater part of the day in the dark, ill-ventilated dwellings of the poor, where the dust in many instances contains tubercle bacilli derived from human sufferers from

[1] *Lancet*, 1898, ii. 1662.

tuberculosis. We have seen that it is by air containing such bacilli finding entrance to the lungs that children are generally infected, but we can easily understand how occasionally milk might be so placed as to receive more bacilli than a child's lungs.

There is another way by which children may become infected by the alimentary tract without deriving the disease from cows. The free intercourse of women of the humbler classes, and the attention they pay to one another's babies, introduces an element of risk. The child may be kissed by a phthisical woman, or may have a spoonful of sweetened liquid given to it that has been previously tasted by such a person.

Tuberculous disease in cows may be the same disease as in man, but it does not follow that the tubercle bacilli present in the milk of diseased animals are very virulent towards man. If small-pox in cows assumes a very mild form, in man it might be argued that tuberculosis possibly does also, and that if only a mild attack of tuberculosis protected against the more severe forms of the disease, the best way to protect the younger members of the community would be to feed them on tuberculous cows' milk.

I am not aware, however, that there is yet proof that the tuberculous disease of cows is capable even of communicating a mild form of tuberculosis to man. We cannot, of course, test the power of infected milk on babies, as can be done in guinea-pigs, but Dr. Emmett Holt[1] tells an interesting story that has some bearing upon the point, and may be looked upon as an extensive experiment innocently performed. Near a large American city was a stock farm of Jersey cows, which supplied milk to a large number of the wealthiest families in the city for a period of ten years. At the end of that time, 45 per cent. of the cows were found by the tuberculin test to be tuberculous. They were killed by order of the State Board of Health, and the diagnosis confirmed in every instance by necropsies. Of the many hundreds of children who had taken the milk, in only one could it be found that tuberculosis had developed, and in that case it was not clear that the milk was responsible. The employés about the farm had also been accustomed for several years to drink skimmed milk in large quantities as a beverage in the place of water. Not one, however, developed tuberculosis.

Such a tale does not lend much support to the view of Dr. Delépine, that "the danger from a contaminated milk-supply is appalling in its magnitude." Much as I respect the ability and judgment of the probable author of another statement which appeared recently in one of our local newspapers,[2] I think it rather premature to tell the public that "it is probable the germ of consumption is carried to us most frequently by meat or milk." That milk is a source of danger in many ways none of us doubt.

[1] *Loc. cit.* [2] *Western Daily Press*, Jan. 30, 1899.

When one learns that as many as 60,000 micro-organisms have been found in a cubic centimetre of milk, one cannot lay too great stress upon the importance of sterilisation. But to arrive at the decision that milk is the most important factor in the spread of tuberculosis appears to me to be an error. If we turn our attention, and that of the public, too strongly in some new direction, we are apt to lose sight of other facts already well established.

There can be no two opinions as to the importance of fresh air and sunlight in destroying the vitality of the tubercle bacillus. Careful regulation of the milk-supply may possibly do a little towards diminishing the death-rate from this disease, but more sunshine and ventilation in the dwellings of the poor will do a great deal more.

(In a recent paper[1] Dr. Leonard Guthrie similarly argues against tuberculous milk being a common cause of tuberculosis in children).

THEODORE FISHER.

